# Efficient Channel Allocation using Matching Theory for QoS Provisioning in Cognitive Radio Networks

**DOI:** 10.3390/s20071872

**Published:** 2020-03-27

**Authors:** Muddasir Rahim, Ahmed S. Alfakeeh, Riaz Hussain, Muhammad Awais Javed, Atif Shakeel, Qadeer ul Hasan, Adeel Israr, Alhuseen Omar Alsayed, Shahzad A. Malik

**Affiliations:** 1 Department of Electrical and Computer Engineering, COMSATS University Islamabad (CUI), Islamabad 45550, Pakistan; muddasir1994@yahoo.com (M.R.); rhussain@comsats.edu.pk (R.H.); atif_shakeel@comsats.edu.pk (A.S.); qadeer.hasan@comsats.edu.pk (Q.u.H.); adeel.israr@comsats.edu.pk (A.I.); smalik@comsats.edu.pk (S.A.M.); 2 Department of Information Systems, Faculty of Computing and Information Technology, King Abdulaziz University, Jeddah 21589, Saudi Arabia; asalfakeeh@kau.edu.sa; 3 Deanship of Scientific Research, King Abdulaziz University, Jeddah 21589, Saudi Arabia; aoalsayd@kau.edu.sa

**Keywords:** channel allocation, cognitive radio network, matching theory, quality-of-service (QoS)

## Abstract

The focus of research efforts in cognitive radio networks (CRNs) has primarily remained confined to maximizing the utilization of the discovered resources. However, it is also important to enhance the user satisfaction in CRNs by finding a suitable match between the secondary users and the idle channels available from the primary network while taking into consideration not only the quality of service (QoS) requirements of the secondary users but the quality of the channels as well. In this work, the Gale Shapley matching theory was applied to find the best match, so that the most suitable channels from the available pool were allocated that satisfy the QoS requirements of the secondary users. Before applying matching theory, two objective functions were defined from the secondary user’s perspective as well as from the channel’s perspective. The objective function of secondary users is the weighted sum of the data rate of the secondary users and the probability of reappearance of the primary user on the channel. Whereas, the objective function of the channel is the maximum utilization of the channel. The weight factors included in the objective functions allow for diverse service classes of secondary users (SUs) or varying channel quality characteristics. The objective functions were used in developing the preference lists for the secondary users and the idle channels. The preference lists were then used by the Gale Shapely matching algorithm to determine the most suitably matched SU-channel pairs. The performance of the proposed scheme was evaluated using Monte–Carlo simulations. The results show significant improvement in the overall satisfaction of the secondary users with the proposed scheme in comparison to other contemporary techniques. Further, the impact of changing the weight factors in the objective functions on the secondary user’s satisfaction and channel utilization was also analyzed.

## 1. Introduction

Radio spectrum is a limited resource and the recent growth of wireless communication has increased the demand for frequency spectrum, which has resulted in congestion. There are two ways to deal with frequency congestion, either: (i) expand the radio spectrum, which is an expensive solution, or (ii) use the white spaces, i.e., spectrum holes, in the existing allocated band and improve the spectrum utilization [1]. The second option is not only cheap but also readily adaptable. When a primary user is not using the channel at a specific time or space, it creates an opportunity of communication for a secondary network. The opportunistic use of the spectrum holes is achieved through a system called cognitive radio network (CRN) [2].

In a CRN, secondary users (SUs) opportunistically share the unused portion of primary band [3]. In a CRN cycle, the process begins with spectrum sensing, in which the spectrum holes, created by the absence of primary users (PUs), are discovered. Accuracy is the key performance indicator for a spectrum sensing scheme, however, quick and efficient sensing is also very important in a CRN as resources are consumed in discovering the holes. Once the spectrum holes are discovered and identified, the opportunities are shared among the SUs. Spectrum sharing includes spectrum access, spectrum allocation, and power allocation. SUs access the idle channels in an interweave, underlay, or overlay transmission mode [4,5]. When there is no PU active, i.e., a spectrum hole is created, the SUs access the channel without causing interference to the PU in the interweave access method. In the underlay scheme, SUs access the channel in the presence of PU simultaneously by using power control, such that the SUs transmit at low power to maintain interference level within bounds. In interweave and underlay modes of communication in CRN, the primary network is oblivious to the presence of the secondary network. In the third option, i.e., overlay mechanism, there is a coordination between the primary network and the secondary network; the SUs access the channel after getting permission from the primary network. Whatever the mode of access in a CRN, on the reappearance of the PU or any possibility of the hazard of interference to the PU, the channel has to be vacated by the SU. This is spectrum mobility or spectrum handoff in a CRN.

In the spectrum handoff, when the PUs reappear, the SUs leave the channel and look for another channel, if available, to complete its unfinished transmission. The selection of a new channel depends on the channel availability and capacity when a handoff decision is made [6,7,8]. Spectrum handoff in a CRN may also be required when the SU experiences the degradation of the quality of service [9,10].

After discovering the vacant channels from the primary network, one of the major challenges in a CRN is channel allocation to the SUs so that the spectrum utilization is maximized. Channel allocation is a critical functionality that is required not only for initiation of the communication but also for handoffs. In a CRN, spectrum handoffs can be frequent as a secondary user has to vacate the channel whenever a primary user reappears; this leads to the requirement of allocating another idle channel to the secondary user. As opportunities are discovered in CRN, it becomes imperative to use them in an optimal fashion. Allocating any channel from the available pool of channels to any SU, i.e., CRN user, in a random fashion may not be the best choice as different applications have different quality of service (QoS) requirements, e.g., some are delay-intolerant (error-tolerant) while others are delay-tolerant (error-intolerant). If there are two channels available in a CRN; one with higher bit error rate than the other and the other channel having a higher probability of reappearance of primary than the first one, then it would be a better choice to assign the channel with low error rate to a SU running a delay-tolerant application and a channel with a low probability of primary reappearance to a SU running a delay-intolerant application.

In this work, we have developed a channel allocation scheme for secondary users in a CRN based on the Gale–Shapley matching algorithm. The proposed channel allocation scheme, the “secondary proposed deferred acceptance (SPDA)” enables us to find an optimal match for the SUs contending for channels from the available channels while taking into consideration the QoS requirements of the SUs and quality of the channels. The performance of the proposed schemes was evaluated in terms of “SU satisfaction”, which is the measure of a SU getting the most favorable channel from the available pool. This maximizes the throughput of the secondary network with minimum interference to the primary network.

The rest of the paper is organized into the following sections. In Section 2, background and related work are discussed. The proposed channel allocation scheme, the “Secondary Proposed Deferred Acceptance (SPDA)”, is presented in Section 3. In Section 4, simulation results and analysis are presented, and finally conclusions are drawn in Section 5.

## 2. Background and Related Work

CRN users access the channels in the absence of the primary users, or with restricted power can go into the underlay mode of communication. Channel allocation is a highly critical function in a CRN where SUs have to switch channels due to frequent spectrum handoffs necessitated by the reappearance of PUs or high levels of interference.

Spectrum handoff is an important part of a CRN for dynamic spectrum access. Spectrum handoff in CRN is mainly classified into two main categories: 1) non-channel switching, also known as non-handoff and 2) channel switching spectrum handoff. In non-channel switching, when a PU reappears, an SU stays on the same channel and waits for the PU to vacate the channel to resume its unfinished transmission [11,12]. For short PU data transmission, the non-handoff mechanism is a better choice, because PU interference is very low [13]. In channel switching handoff, the SU leaves that channel and switches to a new idle channel when the PU reappears. Channel switching handoff can be accomplished in a reactive or proactive manner [11].

In a reactive handoff, the SU reacts to the reappearance of the PU, so it is only after the reappearance of the PU that the SU ceases its transmission on the current channel and searches for a new channel to move the current session [14]. In this approach, sensing delay has a major contribution to the overall handoff delay. Hence, large sensing time leads to a large handoff delay. However, the advantage of the reactive handoff technique is that SU has updated information about the target channel and there are no prediction errors.

Spectrum sensing is performed before the handoff is required in the proactive handoff. This saves the spectrum sensing time, so SUs can immediately switch to the pre-determined target channel [15,16,17]. In the proactive handoff, the target channel is selected based on traffic statistics, such as the probability-based method [18] and the Markov decision process [19]. Hence, the handoff delay is reduced as compared to that in reactive handoff, however, there is a possibility of prediction errors. For example, if the prediction for the PU traffic model goes wrong, the target channel may not be available at the actual time of spectrum handoff, which leads to a miss-detection and false alarm.

Game theory and matching theory have recently been used for channel allocation and spectrum handoff in cognitive radio networks (CRNs); a cognitive channel allocation game has three parts, namely, (i) the players, (ii) the actions, and (iii) the objective function [20]. In a CRN, the players are SUs and PUs and sometimes a set of channels. Each player has an objective function, which targets to maximize the spectrum efficiency. The players take collective actions to achieve the objectives.

Stable matching for channel allocation in a CRN was first presented in [21], using the Gale–Shapley algorithm [22] and considering the users and the channels as men and women, respectively, and utility function as a preference list. There are two types of users, either roaming or non-roaming. Roaming users try to get the best channel from the pool of available channels, but only channels not tried before. Non-roaming users continue transmission on the same channel that was used in the previous CRN cycle. The one-to-one matching problem is formulated between the vacant primary channels and SUs. The data rate is considered to be part of the objective functions of both channels and SUs.

In [23], the authors explained three different matching techniques: (i) canonical matching, (ii) matching with externalities, and (iii) matching with dynamics. Application of matching theory in heterogeneous small cell networks, device-to-device communication, and cognitive radio networks are discussed. However, deferred acceptance (DA) with canonical matching is proposed for CRN. Canonical matching is used in spectrum management within a cell. Therefore, canonical matching with DA is applicable in CRN, in which one must assign orthogonal PU channels to a number of SUs. The result shows improvement in SUs sum rate as compared to random spectrum allocation and classical DA algorithm.

Authors in [24], proposed a self-scheduled multichannel cognitive-radio MAC (SMC-MAC) protocol in which the cognitive users use channel sensing and random contention mechanism for slot selection. For the case, when the SMC-MAC protocol uses only a few contention slots to overcome contention, significant collisions among cognitive users take place. On the other hand, if a large number of contention slots are used for contention mitigation, the collisions are reduced at the expense of low throughput. Another drawback of the SMC-MAC protocol is that if a cognitive user’s transmission results in collision, it has to wait for the next cycle to continue its transmission.

Authors in [25] present the primary proposed deferred acceptance (PPDA) algorithm to achieve stable matching. For channel allocation, the many-to-one matching game model is used. In PPDA, PU proposes its best choice SU according to the preference list, whereas the SU either accepts or rejects the proposal according to its preference criteria. PUs preference list is organized based on the product of the interference caused by the SUs and the amount of fee. SU calculates its preference list according to the achievable throughput (or data rate). Results compare maximal social welfare, maximal PUs utility, maximal SUs utility, stable matching, and random spectrum allocation. For small number of SUs, maximal social welfare performance is better as compared to other techniques. However, when the number of SUs is increased, the stable matching and social welfare performance are almost identical, but the computational complexity is higher for maximal social welfare, maximal PUs utility and maximal SUs, and utility as compared to that for the stable matching. The main reason for higher computational complexity is that 0–1 integer programming-based optimization is used in maximal social welfare, maximal PUs utility, and maximal SUs utility.

Based on the deferred acceptance (DA) algorithm, one-to-one and the one-to-many matching scheme were investigated in [26]. In one-to-one matching only, one PU collaborates with one SU resulting in a better gain for SUs. However, in one-to-many matching, each PU may collaborate with more than one SUs, which leads to better performance for PU as it gets multiple options to find the match and associate with multiple SUs. SU’s satisfaction decreases due to the assignment of multiple SUs to one PU channel.

In [27], the authors proposed a self-organized and distributed model for the channel allocation of licensed channels to the SUs by modeling it as a matching game problem between primary and secondary users. SUs use the logarithm of a posteriori probability based on the presence or absence of PU and rank SUs accordingly. PUs rank their preference list according to the price (or reward) proposed by the SUs. The proposed model shows improvement compared to DA and random allocation. In terms of SUs sum rate, the proposed algorithm provides improvement up to 20% and 60% as compared to the sum rate in DA algorithm and random spectrum allocation, respectively. PUs’ payoffs in this proposed channel allocation scheme are improved by 25% in comparison to that in the DA algorithm.

SUs are the visitors to the vacant channel of the PUs [28]. SUs need to switch from one channel to another available channel when the PU reappears, i.e., a spectrum handoff is necessitated. In [29], authors demonstrated a congestion game based reactive spectrum handoff scheme for a CRN. The quality of service (QoS), price, and handoff cost of the selected spectrum was used to calculate the objective function. Each SU selected a different channel by determining the list of available channels for the spectrum handoff. Thus, this spectrum handoff scheme prevents congestion in the channels by selecting the vacant channel with due consideration of the objective function.

Authors in [30] presented the game theory approach for post handoff target channel sharing. Game theory was used to examine the behavior of SUs by requiring the SUs to share its channel to the other users. Nash equilibrium (NE) and the Nash bargaining solution (NBS) were found for non-cooperative and cooperative users, respectively. The scope of this work was limited as it considers a single channel with two players.

In [31], the authors examined pre-matching and matching in CRN. In the pre-matching phase, maximal SU and channel utilities were obtained. Two utility functions were defined; one for channel and another one for the SU. Channel utility is a quasi-concave function of the SU utility, so a trade-off is involved between SU’s objective function and channel’s objective function. Spectrum efficiency (SE) is the main concern for a SU in a CRN as SU needs to buy the channel or compete when the channel is vacant. Therefore, SU wants to maximize the throughput to save the cost. For a channel rate (T) and available bandwidth (W), the spectrum efficiency is defined as SE=(T×Pr[C≥T]/W) [32], where Pr[C≥T] is the probability of the achieved Shannon’s capacity *C* greater than the channel rate *T*.

## 3. SPDA: Secondary Proposed Deferred Acceptance

In this work, we have proposed “SPDA—secondary proposed deferred acceptance”, a channel assignment scheme in which SUs requiring transmission resources are matched to the most favorable channels taking into account the QoS requirements of the SUs and the channel conditions.

Building upon the existing works of [21] and [23,25,26,27], we defined the objective functions of SUs and channels and construct a preference list. Contrary to [21], we have separate objective functions for SUs and channels. In PPDA scheme [25], since PU proposes and SU accepts or rejects the proposal according to its preference list, the PU’s satisfaction by virtue of increased rewards was improved; however, it was at the cost of deteriorated SU’s satisfaction. Compared to [25], SPDA allows the SUs to propose for matching with the channel that leads to improved SUs’ satisfaction. In contrast to [26], the proposed scheme was based on one-to-one matching without cooperative spectrum leasing. As opposed to [27], where PU’s utility was assumed to be dependent on the SUs proposal, i.e., the rewards, we propose a matching algorithm with objective functions for both the SUs and the channels.

We define two objective functions, one for SUs and another for the idle channels. For any SU-channel pair (m,k), the measure of preference of the *m*th SU for the *k*th channel is the maximal objective function of SU and the measure of preference of *k*th channel for *m*th SU is the maximal objective function of the channel. The preference list is based on the maximal objective of SUs and channels. The Gale–Shapley matching algorithm was used to find SU-channel pairs according to preference order.

### 3.1. System Model

**Figure 1:**
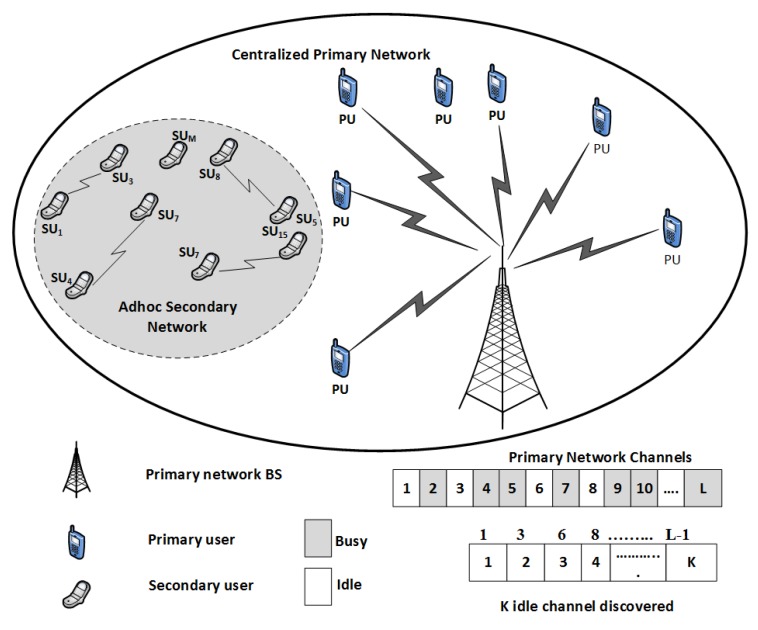
System Model.

We considered an ad hoc cognitive radio network, where there was no central entity to govern the SUs. The SUs can access the idle portions of the spectrum and must leave the channel as PU becomes active on a channel, i.e., PUs hold the right to use the licensed spectrum. A set of SUs $S=\{S_{1},S_{2},\ldots ,S_{M}\}$ has *M* members and the set of PUs $P=\{P_{1},P_{2},\ldots P_{N}\}$ has *N* members as shown in Figure 1. The set of channels of the primary network has a cardinality of *L*, whereas, the set of vacant channels $C=\{C_{1},C_{2}\ldots ,C_{K}\}$, i.e., channels available to the SUs have a varying cardinality of K=|C|, where, (0≤K≤L), which depends on the load on the primary network. The detail list of notations used in this paper is presented in Table 1.

### 3.2. Frame Structure

**Figure 2:**
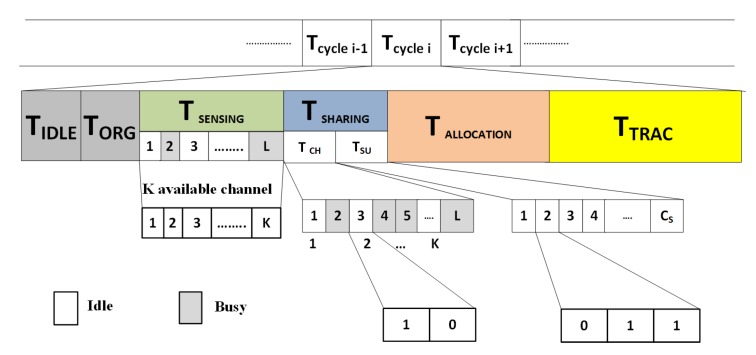
Frame Structure.

The CRN is considered a time-slotted system, where after the idle time $T_{IDLE}$, there are five phases: (i) organization (ii) sensing, (iii) sharing, (iv) allocation, and (v) transmission, as shown in Figure 2. An idle slot at the beginning provides synchronization among the SUs in an ad hoc CRN. After the synchronization, the SUs acquire their IDs in the range of 1 to system capacity $\left(C_{s}\right)$ in the organization phase as in [33]. The sensing of the spectrum to find spectrum holes is performed in $T_{SENSING}$. This information is shared among the SUs during the first sub-phase of sharing, i.e., $T_{CH}$. $T_{CH}$ has sub-slots equal to the number of channels in the primary network. Each of these sub-slots in the $T_{CH}$ has two bits. The first bit represents whether the channel has been sensed or not sensed and the second bit shows the channel state as busy or idle. If the first bit is 1, it indicates that the channel has been sensed by the SU while 0 shows channel has not sensed; the bit value 1 in second-bit position shows channel busy while 0 indicates the idle channel, as shown in Table 2. In the second sub-phase $T_{SU}$ of $T_{SHARING},$ the SUs share their channel utilization information among SUs. In this sub-phase there are $C_{s}$ slots. Every user uses the slot corresponding to its ID to share this information. In our simulations we have kept this value to be 3 bit however the size slot of $T_{SU}$ can be adjusted according to the desired level of precision. These three bits of each sub-slot of $T_{SU}$ sub-phase represent the channel utilization offered by respective SU. If all the three bits are one it indicates maximum channel utilization, i.e., 100% and when the value of these three bits is 0 then the channel utilization is below 40%. We divide this information into eight levels using the three-bit as shown in Table 3. The channel allocation is performed in $T_{ALLOCATION}$ using the Gale–Shapley algorithm. The actual utilization of the channel for data transmission is after the allocation of a channel to a SU, where it transmits the data in $T_{TRAN}$.

### 3.3. SU Objective Function

In this section, we formulate the objective of each SU with the corresponding channel and denote it as $O_{SU}^{\left(i,k\right)}$ as i=1,2…M and k=1,2,…,K. An SU in a CRN is mainly concerned with the throughput and the probability of reappearance of PU as SUs need to pay for bandwidth or compete for the vacant channel. We define the throughput of *i*th SU for *k*th channel as,

(1) $$\begin{array}{c} R^{\left(i,k\right)}=W\times log_{2}\left(1+\frac{P_{SU}\times h_{SU}^{i,k}}{N_{o}}\right) \end{array}$$

where *W* is available bandwidth, $P_{SU}$ is the transmission power of SU, $h_{SU}^{i,k}$ is the channel gain, and $N_{o}$ is channel noise.

The other factor that contributes to the objective function of a SU is the probability of reappearance of PU on the *k*th channel. The reappearance of PU on a channel is determined through traffic statistics, such as the probability-based method [18]. The secondary user keeps monitoring the primary activity on the current channels and leaves it on the reappearance of the PU. By combining the throughput and probability of reappearance of PU $p_{r}$ on the channel we define the objective function of *i*th SU for *k*th channel as:

(2) $$\begin{array}{c} O_{SU}^{\left(i,k\right)}=\alpha \times R^{i,k}+\left(1-\alpha \right)\times p_{r}^{k} \end{array}$$

where α is a weight factor and 0≤α≤1. By changing the value of weight factor α, different classes of services can be provisioned. An SU with delay intolerance (i.e., real-time and noise-tolerant) application will choose a large value of α, whereas an SU with delay tolerance (i.e., non-real time but noise-intolerant) application will choose a small value of α. Using the objective function, the preference value $O_{SU}^{\left(i,k\right)}$ of *i*th SU for *k*th channel is determined and a preference list is constructed in a descending order. The channel with the highest preference for the respective SU is at the top of the list and the one with the least at the bottom. Combined preference lists of all the SUs gives us a matrix of order M×K.

### 3.4. Channel Objective Function

Sharing of available *K* channels among the *M* SUs also depends on the channel objective function. From the channel’s perspective, it is desirable to maximize its utility (in terms of percentage utilization and offered price), thus a channel objective function is defined as:

(3) $$\begin{array}{c} O_{CH}^{\left(i\right)}=p_{U}^{i} \end{array}$$

Each SU shares its offered utility for the channel and a preference list $P_{CH}^{\left(i\right)}$ is constructed, where at the top of this list is the SU offering the highest utility and at the bottom of the list is the SU offering lowest utility for the channel.

### 3.5. SPDA Algorithm

**Figure 3:**
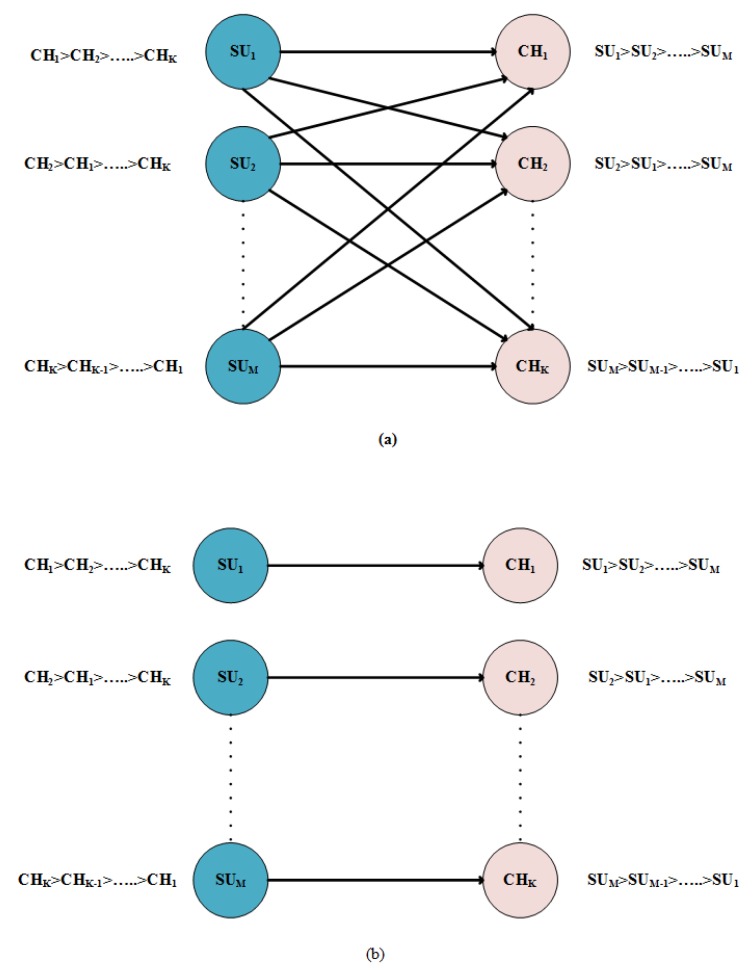
SPDA Channel allocation: (**a**) before allocation, (**b**) after allocation.

We used the Gale–Shapley algorithm to find the optimal matching. The algorithm, shown in Algorithm 1, is expressed as a sequence of attempts from SUs to channels. At any time during the process, a channel is either allocated to the SU or remains free. Each SU proposes the highest priority channel in its preference list until a channel is allocated. An SU rejected by a channel can not re-attempt the same channel. When a user proposes a free channel, the channel will immediately accept its proposal; whereas, if the user proposes an already engaged channel, the channel compares the new and current users and gets engaged to a more favored among the two users. That is if the channel prefers the current user, the proposal from the new user is rejected. But if the channel prefers the new requester, the channel breakdowns the engagement with the current user and accepts the new proposal. The process continues until all SUs are engaged or have exhausted all of their options. At the conclusion, the matching between SU and channel serves as an allocation mechanism as shown in Figure 3.
**Algorithm 1:** Secondary proposed deferred acceptance (SPDA) algorithm for channel allocation.
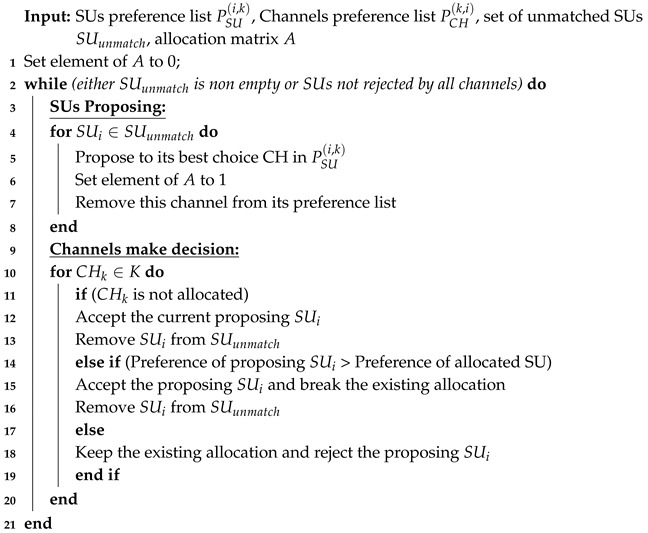


### 3.6. Use Case

**Figure 4:**
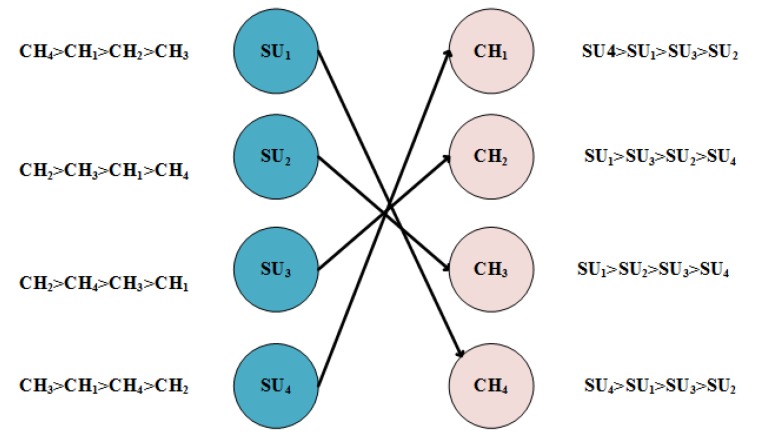
Use case.

To elaborate the algorithm, consider a set of hypothetical preferences for SUs and channels. Table 4 shows the preference list of users and channels’ preferences are tabulated in Table 5; the users target the channel according to higher priority $SU_{1}$, $SU_{2}$, $SU_{3}$, and $SU_{4}$ target $CH_{4}$, $CH_{2}$, $CH_{2}$, and $CH_{3}$, respectively; only $CH_{2}$ is targeted by two users, so channel checks its preference list for accepting the proposal. $SU_{3}$ is more favorable than $SU_{2}$ as $SU_{3}$ is higher on priority for channel. So $CH_{2}$ accepts $SU_{3}$ and rejects $SU_{2}$. At this stage, $SU_{1}$, $SU_{3}$ and $SU_{4}$ are engaged and only $SU_{2}$ is still free. So, $SU_{2}$ proposes to the next channel in its preference list, which is $CH_{3}$. As $CH_{3}$ is already engaged to $SU_{4}$, it weighs both of its options—the new proposal and the one it is already engaged to. As per Table 5 of channel preference list, channels prefers $SU_{2}$ over $SU_{4}$, so $CH_{3}$ accepts the new proposal and breakdowns with $SU_{4}$. Now, $SU_{4}$ is free, $SU_{4}$ attempts for $CH_{1}$, which is already free, so its proposal is accepted straight away. This leads to a stable match as shown in Figure 4 $\left(SU_{1},CH_{4}\right)$, $\left(SU_{2},CH_{3}\right)$, $\left(SU_{3},CH_{2}\right)$, and $\left(SU_{4},CH_{1}\right)$.

### 3.7. SU Satisfaction

SU’s satisfaction ($SU_{sat}$) is determined through its allocated channel in its preference list. For example, if an SU gets its highest preferred channel, we assume that SU satisfaction is 100%. In our system model, we have *K* number of channels and *M* number of SUs, so if an SU gets allocated its *x*th preferred channel, the $SU_{sat}$ is:

(4) $$\begin{array}{c} SU_{sat}=\frac{(K+1)-x}{K} \end{array}$$

The average $SU_{sat}$ in CRN having *M* number of SU is:

(5) $$\begin{array}{c} \bar{SU_{sat}}=\frac{\sum_{i=1}^{M}\left(K+1\right)-x_{i}}{K\times M} \end{array}$$

For example, we have K=4 and M=4 and each SU gets its 1st preferred channel, the average SU satisfaction is calculated using Equation (5) as $\bar{SU_{sat}}=\frac{(4+1)-1+(4+1)-1+(4+1)-1+(4+1)-1}{4\times 4}=1$, i.e., the average SU satisfaction is 100%. However, if two SUs get their 1st, one SU gets its 2*^nd^* and one SU gets its 3rd preferred channel, the average SU satisfaction will be; $\bar{SU_{sat}}=\frac{(4+1)-1+(4+1)-1+(4+1)-2+(4+1)-3}{4\times 4}=0.8125$, i.e., the average SU satisfaction is around 81%.

### 3.8. Channel Utilization

Channel utilization ($CH_{ut}$) defines the portion of the available time on a PU vacant channel before reappearance of the PU being used by SU for its transmission. We have *M* number of SUs, so if a channel gets allocated its *y*th preferred SU, the $CH_{ut}$ is:

(6) $$\begin{array}{c} CH_{ut}=\frac{(M+1)-y}{M} \end{array}$$

The average $CH_{ut}$ of CRN having *K* number of channel is:

(7) $$\begin{array}{c} \bar{CH_{ut}}=\frac{\sum_{k=1}^{K}\left(M+1\right)-y_{k}}{M\times K} \end{array}$$

## 4. Simulation Scenario and Comparison

In this section, performance evaluation of the proposed SPDA matching is presented and compared with the SMC-MAC and the PPDA protocols. We considered a primary network consisting of 10 PUs and a CRN with *M* number of SUs, varying from 2 to 20. Out of *L* primary channels, there were *K* free channels available to the CRN. The detailed list of simulation parameters is provided in Table 6. We performed a Monte–Carlo simulation and results were averaged over $10^{6}$ experiments.

We implemented the following protocols including our proposed scheme.

**SMC-MAC:** In SMC-MAC, available channels are randomly accessed by the SUs in the contention period, which may lead to collisions if more than one SUs contend for the same channel [24].

**PPDA:** In PPDA, PUs propose its best choice of SU according to the preference list, and SUs either accept or reject the proposal according to their preferences, based on the offered price of the channel [25].

**SPDA:** This is the proposed scheme, wherein each SU proposes to the best channels according to its preference list and the channel either accepts or rejects the proposal of the SU, based on the utility offered on the channel.

**Figure 5:**
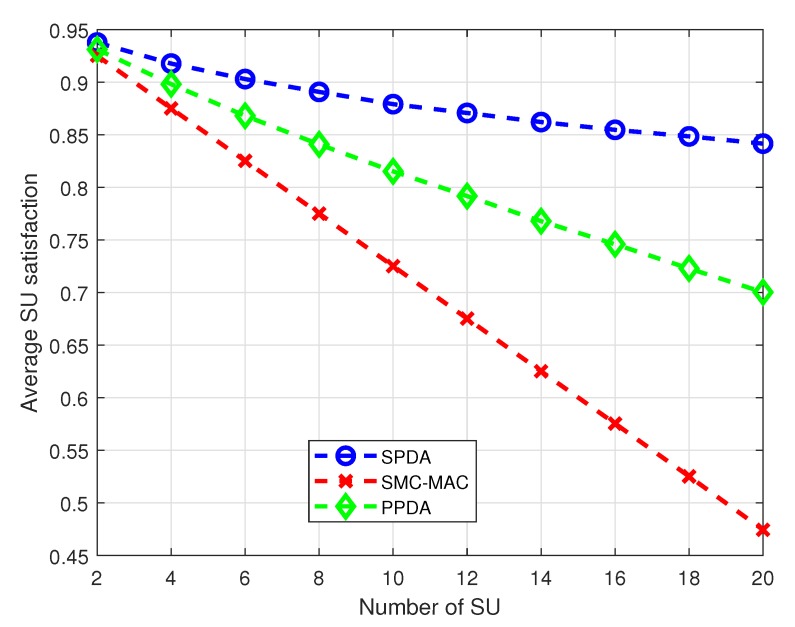
Average SU satisfaction at different numbers of SU.

Figure 5 shows the average SU’s satisfaction with a different number of SUs in the CRN. The proposed SPDA shows a significant improvement in satisfaction compared to SMC-MAC and PPDA. SPDA outperforms both SMC-MAC and PPDA for the whole range of the number of SUs; however, performance improvement is significantly higher with a greater number of SUs in the CRN as depicted in Figure 5. When the number of SUs is 4, SPDA shows approximately 6% and 3% improvement as compared to the SMC-MAC allocation and PPDA respectively. As the number of SU is increased to 20, the SPDA gives approximately 36% and 14% higher satisfaction than the SMC-MAC and the PPDA respectively.

**Figure 6:**
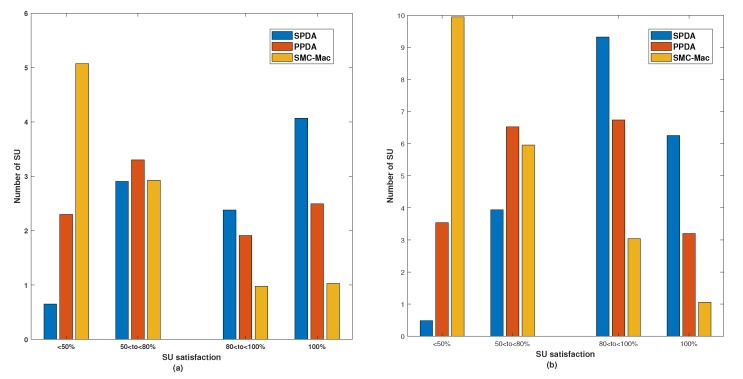
Number of SU at different level of SU satisfaction: (**a**) M = 10, (**b**) M = 20.

Figure 6 shows the number of SUs at different levels of SU satisfaction for two different cases (a) M = 10 in Figure 6a, (b) M = 20 in Figure 6b. In all three schemes, i.e., SMC-MAC, PPDA, and proposed SPDA, for the simulation there were 10 SUs in the CRN, seen in Figure 6a. The highest number of SUs get 100% satisfaction, i.e., getting its most preferred channel from the available pool of channels. Likewise, the number of SUs getting below the average channel is lowest for SPDA. With SMC-MAC, 50% of users get the above-average channel, with PPDA the percentage rises to 75% and with SPDA it is close to 94%. The sum of the individual number of SU is equal to 10. However, Figure 6b shows the similar results for 20 SUs.

**Figure 7:**
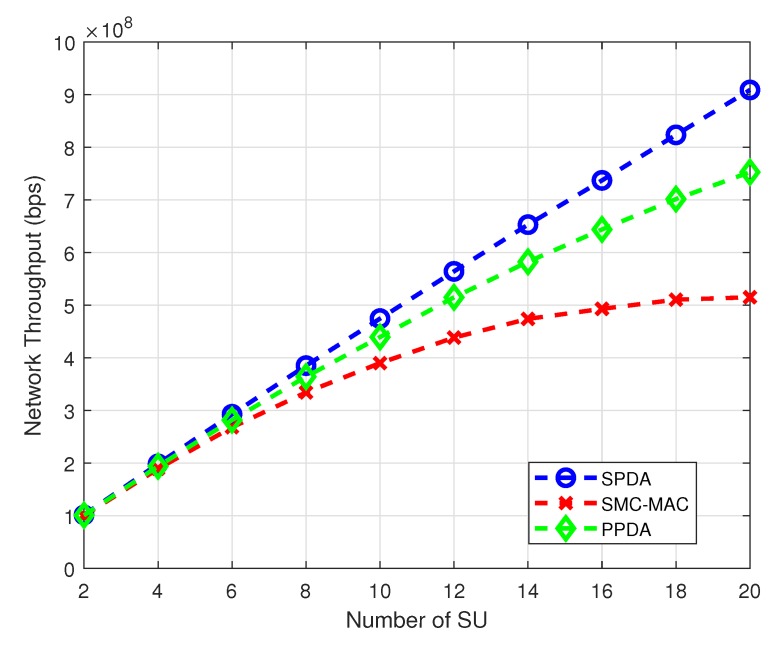
Network throughput at different number of SU.

The network throughput can be increased by optimally utilizing the channels according to the user requirements. Network throughput with variation in the number of SUs demonstrates in Figure 7. With an increasing number of SUs the network throughput also increases for all three schemes. However, for a large number of SUs the SPDA achieves much better network throughput than PPDA and SMC-MAC.

**Figure 8:**
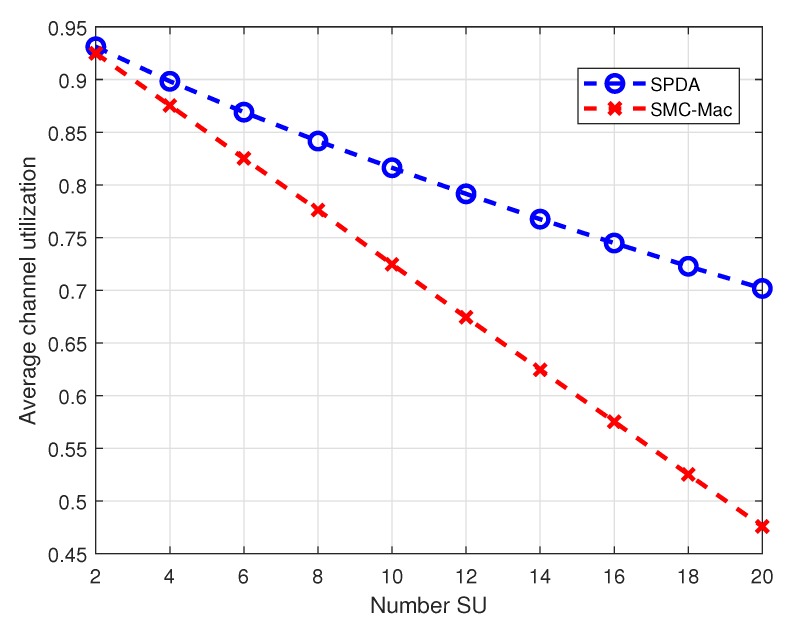
Average channel utilization at different number of SU.

Results in Figure 8 show the average channel utilization with varying number of SUs in the CRN. The performance of proposed SPDA in terms of channel utilization is better than SMC-MAC. For the small number of SUs in the system the channel utilization for both cases is high, exceeding 90% for the number of SU less than 4. When the number of SUs increases from 4 to 20 channel utilization gradually decreases for both schemes, however, it is much smaller steep for SPDA, providing approximately 23% better channel utilization.

**Figure 9:**
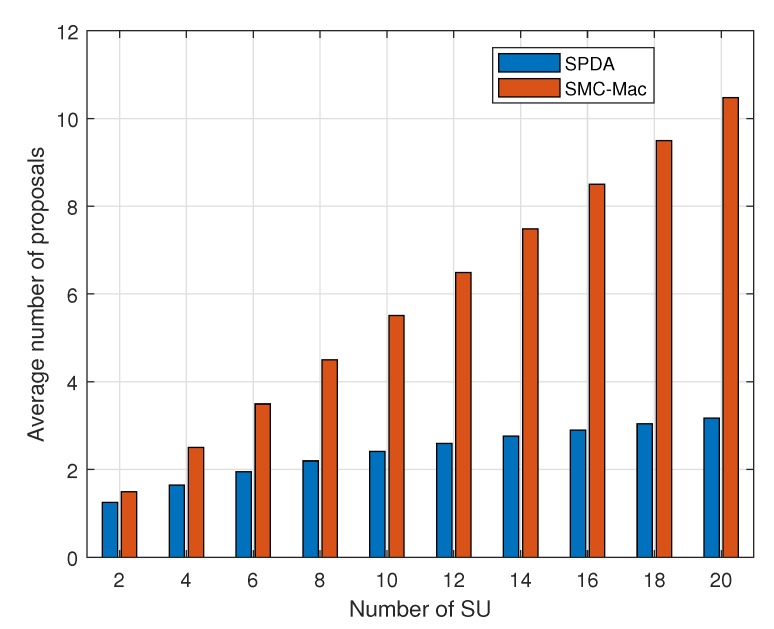
Average number of proposal with a varying number of SUs in cognitive radio networks (CRNs).

Figure 9 compares the average number of proposals before the system is stabilized and the channel allocation is finalized. We compare the proposed SPDA with the SMC-MAC technique and results show that our proposed algorithm outperforms by reaching stabilization earlier. The quick stabilization of the system allows more time for SUs to utilize the channel for data transmission.

**Figure 10:**
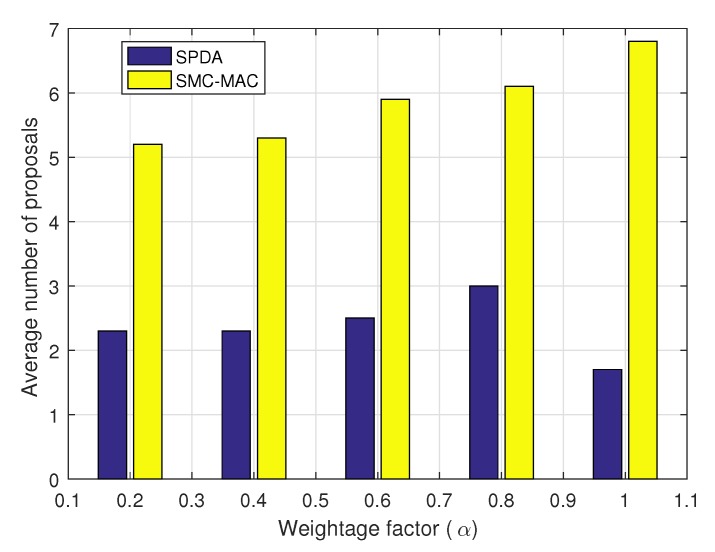
Average number of proposal with variation in the weightage factor, (α).

We have also analyzed the impact of weightage factor α on the average number of proposals required before stabilization. In Figure 10, the α varied from 0.2 to 1. A comparison with the SMC-MAC technique shows that our proposed SPDA algorithm remains better for any value of α.

**Figure 11:**
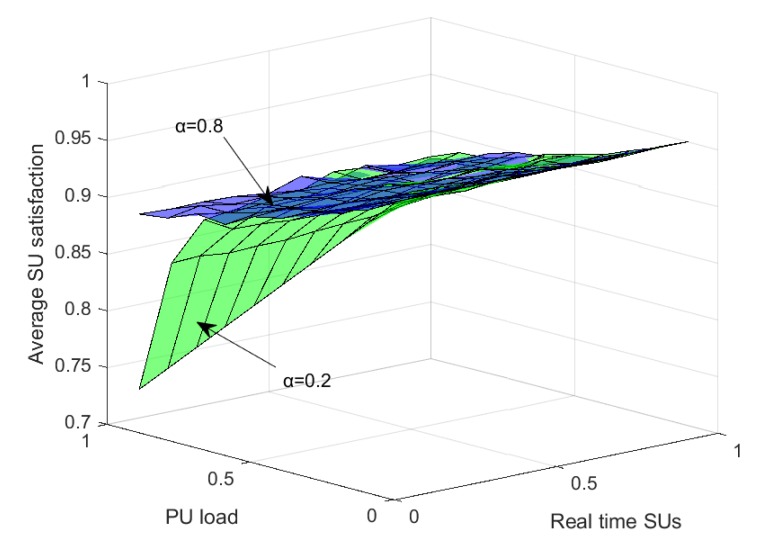
Average SU satisfaction at a different value of α with variation in the PU load and real-time SUs.

It is also interesting to visualize the SU satisfaction with a varying load on the primary network and for different ratios of real-time (i.e., error-tolerant) SUs to delay-tolerant (i.e., error-intolerant) SUs. Figure 11 demonstrates the trend of SUs’ satisfaction for two values of α, i.e., 0.2 and 0.8. For α=0.2, when the PU load increases the SUs’ satisfaction drops rapidly, however, it has a slightly increasing trend with an increase in the ratio of the real-time SUs in the CRN. For α=0.8 the satisfaction remains higher than that at α=0.2 and also the rate of drop in SU satisfaction is much slower with an increase in load on the primary network. Higher satisfaction at a higher value of α is because throughput gets higher weightage than PU reappearance as per the objective function, defined in Equation (2). A higher ratio of real-time SUs means that more SUs are delay-intolerant but error-tolerant and can communicate in the noisy channel. This results in increased average SU’s satisfaction in the network.

**Figure 12:**
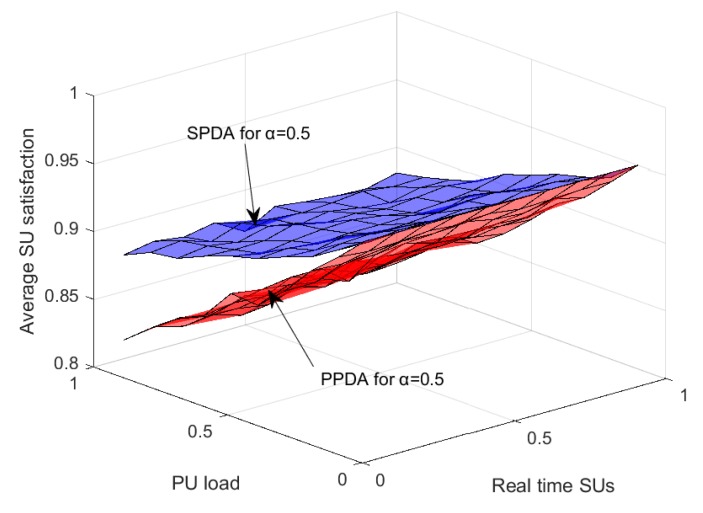
SPDA and PPDA average SU satisfaction with variation in PU load and real time SUs.

SU satisfaction of two schemes are compared in Figure 12, considering the real-time SUs as well as the PU load and the weightage factor α=0.5. When the PU load increases, the SU satisfaction decrease, however, the decrease in the proposed scheme SPDA is much slower than PPDA. SU satisfaction increases when the number of real-time SUs increases for both SPDA and PPDA. Figure 12 shows that the proposed scheme performance is better than PPDA.

## 5. Conclusions and Future Work

In this paper, we proposed a “secondary user proposed deferred acceptance (SPDA)” scheme for channel allocation in a CRN. The Gale–Shapley matching theory was used to create an association between the SU and the channel, which serves the purpose of channel allocation. The goal is to maximize the “SU satisfaction”, considering two different classes of SUs, i.e., delay-intolerant (real-time/error-tolerant) and delay-tolerant (error-intolerant). This matching algorithm targets to allocate the right channel to the right SU based on channel quality and SU class of service requirements. We have compared the proposed SPDA scheme with SMC-MAC channel allocation, where a SU is allocated a channel randomly without consideration of the SU’s QoS requirements and channel quality. SPDA is also compared with “primary proposed deferred acceptance (PPDA)”, where primary user proposes and SU accepts or rejects the offer. Simulation results show that the proposed SPDA scheme can improve the SUs satisfaction up to 15% and 8% as compared to SMC-MAC and PPDA, respectively. Results also show an improvement in the average number of proposals required to finalize the allocation as compared to SMC-MAC. SPDA is SU-optimal, which means the SUs will benefit much more from this matching, however, the PU satisfaction is not optimal. In the future, this work can be extended for optimizing both the “SU satisfaction” and “PU satisfaction” collectively.

## Figures and Tables

**Table 1:** List of Notations.

Description	Parameter
Number of PU	N
Number of SU	M
Number of available channel	K
System Capacity	$C_{s}$
Primary Traffic Load	ρ
Throughput	R
Bandwidth	W
Transmission Power	$P_{SU}$
Channel Gain	$h_{SU}$
Channel Noise	$N_{o}$
Objective Function of SU	$O_{SU}$
Weight Factor	α
Probability of reappearance of PU	$p_{r}$
Channel Objective Function	$O_{CH}$
SU’s Satisfaction	$SU_{sat}$
Channel Utilization	$CH_{ut}$

**Table 2:** Channel status.

Slot	State	Status
First bit	1	Channel sensed idle
Second bit	0	
First bit	1	Channel sensed busy
Second bit	1	
First bit	0	Channel not sensed
Second bit	x	

**Table 3:** Channel utilization.

First Bit	Second Bit	Third Bit	Channel Utilization
1	1	1	100%
1	1	0	above 90%
1	0	1	above 80%
1	0	0	above 70%
0	1	1	above 60%
0	1	0	above 50%
0	0	1	above 40%
0	0	0	below 40%

**Table 4:** SUs preference list.

	Preferences			
	1st	2nd	3rd	4th
SU1	4	1	2	3
SU2	2	3	1	4
SU3	2	4	3	1
SU4	3	1	4	2

**Table 5:** Channel’s preference list.

Channel Preferences			
**1st**	**2nd**	**3rd**	**4th**
SU1	SU3	SU2	SU4

**Table 6:** Simulation parameters.

Parameter	Values
Number of PUs (*N*)	10
Number of SUs (*M*)	2∼20
Number of Primary channel (*L*)	10
Primary traffic load	0∼1
weight factor (α)	0∼1
